# Searching Through Alternating Sequences: Working Memory and Inhibitory Tagging Mechanisms Revealed Using the MILO Task

**DOI:** 10.1177/2041669520958018

**Published:** 2020-10-15

**Authors:** Ian M. Thornton, Todd S. Horowitz

**Affiliations:** Department of Cognitive Science, Faculty of Media and Knowledge Sciences, University of Malta, Msida, Malta; National Cancer Institute, National Institutes of Health, Bethesda, MD, USA

**Keywords:** Multi-Item Localisation task, MILO, Trail Making Test, TMT, visual search, attention, working memory, inhibitory tagging

## Abstract

We used the Multi-Item Localisation (MILO) task to examine search through two sequences. In *Sequential* blocks of trials, six letters and six digits were touched in order. In *Mixed* blocks, participants alternated between letters and digits. These conditions mimic the A and B variants of the Trail Making Test (TMT). In both block types, targets either *vanished* or *remained* visible after being touched. There were two key findings. First, in Mixed blocks, reaction times exhibited a saw-tooth pattern, suggesting search for successive pairs of targets. Second, reaction time patterns for vanish and remain conditions were identical in Sequential blocks—indicating that participants could ignore past targets—but diverged in Mixed blocks. This suggests a breakdown of inhibitory tagging. These findings may help explain the elevated completion times observed in TMT-B, relative to TMT-A.

## Introduction

Recently, we introduced a mobile app version of the Multi-Item Localisation (MILO) task (Thornton & Horowitz, 2020). The MILO task probes the temporal constraints that influence target selection during search through multi-item sequences. Previously, we have used the MILO task to show that when locating a given item in a sequence, both *retrospective* (i.e., where you have been) and *prospective* (i.e., where you need to go next) context within a trial affects search performance (Horowitz & Thornton, 2008; Thornton & Horowitz, 2004).

The goal of this study was to examine what happens to these context effects when a trial contains two interleaved sequences. Such an increase in task demands is an important component of the widely used Trail Making Test (TMT), where interleaving sequences is known to systematically increase overall completion time (Bowie & Harvey, 2006; Lange et al., 2005; Rabin et al., 2007; Reitan, 1958; Salthouse, 2011). Here, we use a new MILO task variant that borrows directly from the TMT, allowing us modulate inherent task demands while mapping out the patterns of responses within interleaved trials. Our primary focus is on further understanding the nature of inhibitory tagging mechanisms thought to operate during MILO and related search tasks (e.g., Klein & MacInnes, 1999; Wang & Klein, 2010). However, we also hope to shed new light on exactly why performance deteriorates when sequences are interleaved, observations that may be of clinical relevance when interpreting TMT costs. We begin by briefly introducing the TMT and the MILO task before presenting our new experimental findings.

### The TMT

The TMT is frequently administered as part of standard neuropsychological assessment and is also a very common research tool (Bowie & Harvey, 2006; Lange et al., 2005; Rabin et al., 2007; Reitan, 1958; Salthouse, 2011). Usually taken as a pen-and-paper test (although see e.g., Fellows et al., 2017; Salthouse & Fristoe, 1995; Woods et al., 2015), it comes in two variants. In TMT-A, participants are asked to quickly and accurately draw lines between numbered circles on a page without lifting their pen. Each circle contains numbers between 1 and 25, and the instruction is to start at the number 1 and proceed in order until reaching the number 25. In TMT-B, the page contains both numbers and letters, and participants are instructed to alternate in order between them, starting at the number 1, followed by the letter A, then the number 2, the letter B, and so on, until reaching the number 13 (see Bowie & Harvey, 2006 for protocol details). The main dependent measure is total time to complete the test—measured with a stopwatch—although error information can also be recorded (e.g., Klusman et al., 1989; Kopp et al., 2015).

Much of the clinical and research interest in this task centres on the fact that TMT-B is considerably more demanding than TMT-A, giving rise to consistently longer completion times. While both variants place demands on visual search, psychomotor skill, and processing speed (see Sánchez-Cubillo et al., 2009 for review), TMT-B is thought to place additional demands on working memory, set-switching, and inhibitory control (Arbuthnott & Frank, 2000; Kortte et al., 2002; Salthouse, 2011; Sánchez-Cubillo et al., 2009). The involvement of these cognitive components has been established by observation of clinical subpopulations (for review, see Lange et al., 2005) and in numerous correlation/regression studies, pairing TMT measures with other well-known tasks (e.g., Arbuthnott & Frank, 2000; Kortte et al., 2002; Salthouse, 2011).

 Although there continues to be debate about precisely which cognitive components underlie TMT-B costs, there appears to be general agreement that it targets the fluid (Salthouse, 2011) or flexible (Kortte et al., 2002) cognitive abilities associated with executive function (Fellows et al., 2017; Sánchez-Cubillo et al., 2009). In this study, our question was whether the need to engage additional cognitive components with interleaved sequences would also influence MILO performance. If so, we hoped that the within-trial resolution and temporal context manipulations available in the MILO task would shed additional light on the nature of the costs involved.

### The MILO Task

We developed the MILO task as a computer-based research tool for exploring the temporal context of visual search (Horowitz & Thornton, 2008; Thornton & Horowitz, 2004). In addition to the iPad app version of the task used here (Thornton & Horowitz, 2020), there is also a cross-platform online version that can be previewed at https://maltacogsci.org/MILO/DEMO/. Both versions of the task, along with the source code, may be freely obtained by contacting the authors.

As in TMT, MILO participants are required to search through a specific sequence of targets, such as the letters A through H, or the numbers 1 through 8 in order. Rather than connecting the elements on paper, MILO responses involve clicking directly on targets with a mouse or touching them on a touchscreen. Importantly, in addition to measuring overall completion time, the MILO task also provides a profile of reaction time patterns across all items in a sequence. This is achieved by having participants complete multiple (e.g., 20), short (e.g., 8 item) trials using either randomly generated novel display layouts or fixed patterns, depending on the research question. The use of multiple trials makes it possible to establish within-participant estimates of the time taken to locate each subsequent item in a sequence, a measure we call serial reaction time (SRT; Horowitz & Thornton, 2008; Thornton & Horowitz, 2004, 2020).

A number of simple manipulations allow exploration of both *retrospective* (i.e., the influence of previous actions on localisation of the current target) and *prospective* (i.e., the influence of future plans on the current target) aspects of search behaviour with the MILO task. For example, in our previous work, we were able to show that participants had almost perfect memory for the locations they had already visited during a trial. We did this by introducing a manipulation in which targets either *vanished* or *remained* visible once selected. The SRT patterns for these two types of trial were essentially identical (Thornton & Horowitz, 2004) indicating very effective inhibitory tagging (Klein, 1988). This tagging process is location-based rather than object-based, as the Vanish and Remain SRT functions separate as soon as either local or global motion is added to the displays (Horowitz & Thornton, 2008).

We have also used the MILO task to demonstrate that participants consistently plan ahead when engaged in sequential search. Such planning is most obvious at the start of a sequence, reflected in highly elevated first response times (Basoudan et al., 2019). However, using a shuffle manipulation, in which the identities of items ahead of the current target swapped positions, we were able to show planning effects influencing SRT patterns up to four items ahead (Thornton & Horowitz, 2004, 2020; see Kosovicheva et al., 2020 for related findings).

### Current Study

In this study we modified the basic MILO task by including two sequences on each trial. These sequences mimic the intrinsic load manipulation of the TMT. Our motivation for studying interleaved sequences was to gain further understanding of the nature of inhibitory tagging during MILO Remain trials (Horowitz & Thornton, 2008; Thornton & Horowitz, 2004, 2020). In previous studies from our group, having participants perform a secondary task while completing MILO trials, such as listening for comprehension (Luffingham, 2013) or retaining a spatial layout in memory (Zammit, 2017), did not lead to any changes in Remain SRTs relative to Vanish trials. The lack of interference suggested that the inhibitory mechanism might be automatic and encapsulated so as not to require high-level cognitive resources.

**Figure 1.**
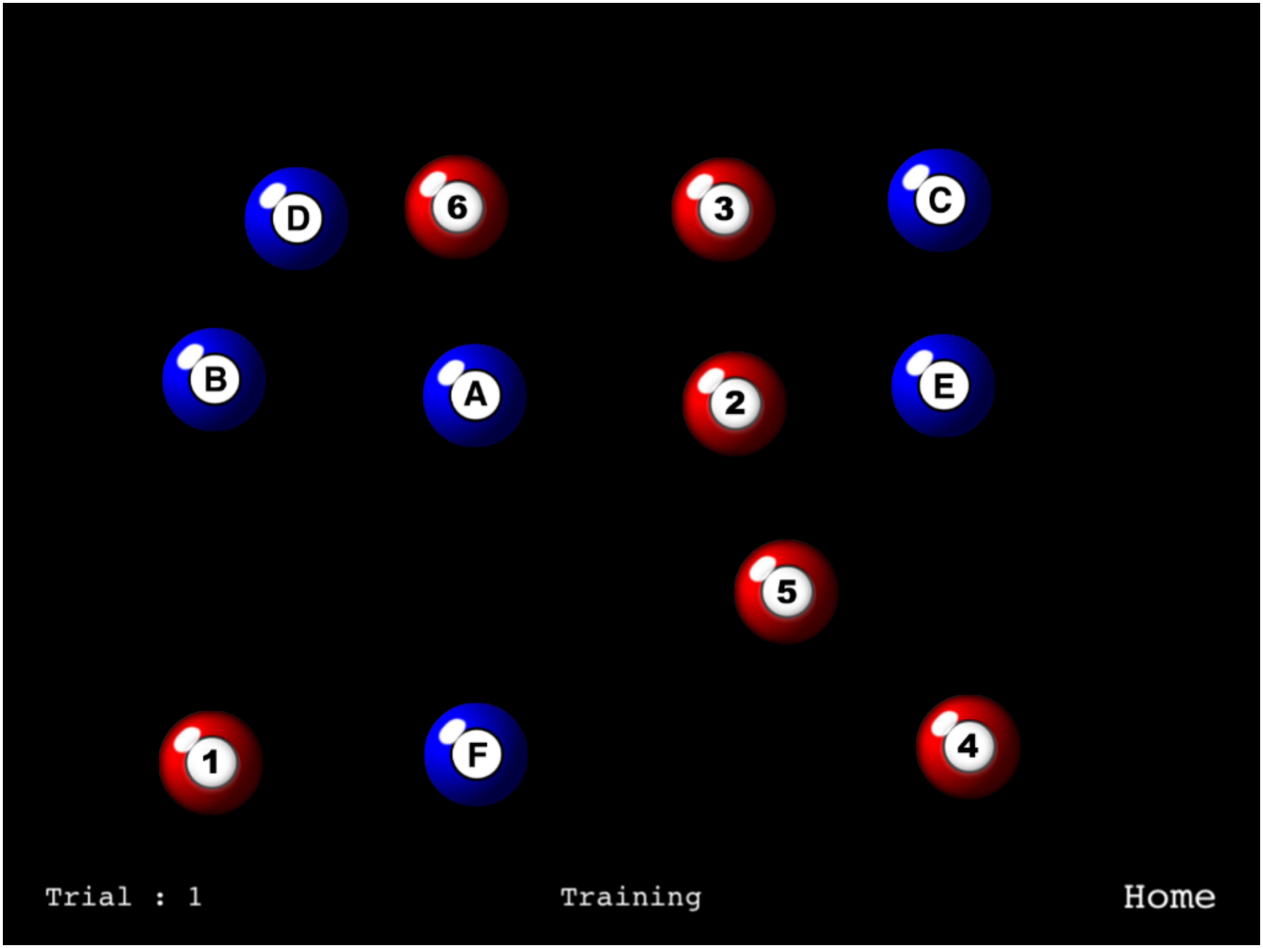
Example screen shot from the milo task with two sequences. In *sequential* blocks of trials, participants touched all of the letters in order before the digits, or vice versa (counterbalanced). In *mixed* blocks, targets from two sequences were interleaved, so the correct order would be A-1, B-2, and so on, or 1-A, 2-B, and so on, again counterbalanced across participants.

Here, we borrowed directly from the TMT and produced an MILO variant that could be performed either with low or high intrinsic load. Figure 1 shows an example display in which there are always 12 items, the letters A–F and the numbers 1–6. During *Sequential* blocks of trials (low load; corresponds to TMT-A), participants were instructed to touch the letters in order, followed by the numbers, or vice versa (counterbalanced). During *Mixed* blocks of trials, the instruction was to alternate between letter and number targets (high load; corresponds to TMT-B).

As with TMT, we expected overall trial completion time to be longer in Mixed blocks compared with Sequential blocks. Note that with MILO, display characteristics are identical in the two block types—with the same items appearing on every trial, albeit in random positions—so any difference in timing would only reflect changes in task difficulty. In Sequential blocks, we expected the SRT patterns to be very similar to those observed in our previous studies, the only unknown being the cost of switching sequences after the sixth response. During Mixed blocks, our question was whether the additional cognitive resources needed to interleave two target types within a trial would interact with the need to inhibit past locations on the Remain trials.

## Methods

### Participants

Twelve participants (10 females; mean age = 24.6 years, standard deviation = 2.5; 2 left handed) from the University of Malta community took part in this study in return for a payment of €10. Sample size was determined prior to data collection. An analysis of 12 previous data sets showed an average observed effect size (partial-eta squared) of 0.72 (standard deviation = 0.2), which indicated a minimum sample size of 9 participants would be sufficient to detect relevant changes in the pattern of SRTs. See Thornton and Horowitz (2020) for further details of this power analysis. Participants were randomly assigned to a group that started each trial with a letter or number, with six participants per group.

All participants reported normal or corrected-to-normal vision. Prior to taking part in the study, participants were given written information about the study, and consent forms which were signed. All methods and procedures conformed to the Ethics and Data Protection Guidelines of the University of Malta.

### Equipment

The stimuli were displayed on an iPad Air (Model A1474) with screen dimensions of 20 × 15 cm (24.6 cm diagonal) and an effective resolution of 1,024 × 768 pixels at 132 ppi. The iPad was placed on a table in front of the participant in landscape mode. As viewing distance could only be approximately estimated at 50 cm, we report stimulus measures in both pixels and degrees visual angle. The MILO Switch app was custom written in objective-C using Xcode and Cocos2d libraries. Source code is available on the Open Science Framework (OSF) page associated with this study at https://osf.io/ugw9n/.

### Stimuli

The stimuli are shown in Figure 1. Characters were drawn in black within the context of red and white (numbers) and blue and white (letters) pool balls, which had shading to provide a slight three-dimensional effect. Each ball had a diameter of 85 pixels and subtended approximately 1.8° visual angle. The 12 targets were positioned randomly on each trial within an invisible 4 × 4 grid that was centred on the screen. Individual targets were randomly jittered by up to 80 pixels horizontally and 30 pixels vertically within the grid to reduce the apparent regularity of the display.

### Procedure

The experiment was run in a sound-attenuated booth under low lighting conditions with no overhead lights, in order to minimise screen glare. Following typical TMT procedure, all participants completed the less demanding Sequential block before the Mixed block of trials. As the Vanish and Remain trials were interleaved, this variation was explicitly shown. The experimental session lasted approximately 30 minutes, with participants completing two blocks of 30 correct trials (each containing 15 Vanish and 15 Remain trials), with the Sequential block always preceding the Mixed block. An error would immediately terminate a trial, which was then automatically replaced with a new random version of the same condition. Based on our previous studies, we expected error rates to be extremely low. We include the average number of error trials per block as part of the data figures below, and the raw data are available in the Supplementary Material. However, this dependent variable was not included in our analysis.

### Data Analysis

To provide consistency with TMT studies, we begin by reporting overall median completion times. These were analysed using a 2 (Block Type: Sequential/Mixed) × 2 (Condition: Vanish/Remain) repeated measures analysis of variance (ANOVA).

To more fully capture within-trial patterns of performance, we report the median SRT for each target position, averaged across all trials completed by each participant in each condition. We first present the data for each type of sequence separately, using the same 2 (Condition: Vanish/Remain) × 12 (Target Item) repeated measures ANOVA, and then, for the sake of completeness, we compare across block type using the full 2 (Block Type: Sequential/Mixed) × 2 (Condition: Vanish/Remain) × 12 (Target Item) repeated measures ANOVA.

Violations of sphericity involving the Target factor were corrected by applying the Greenhouse–Geisser adjustments to the appropriate degrees of freedom. Note that full ANOVA results are provided as Supplemental Material, with text reporting focusing on the findings of interest.

### Data Availability Statement

The raw data and full summary statistics are available on the OSF page associated with this study at https://osf.io/ugw9n/

## Results

**Figure 2.**
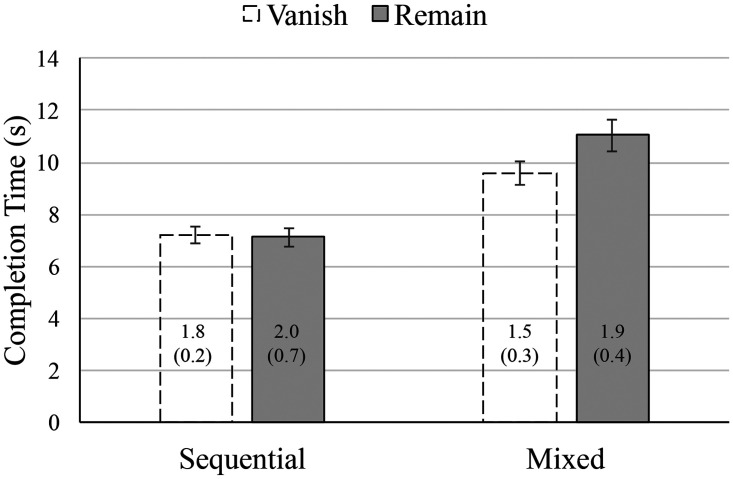
Overall median completion time (bars) and mean number of error trials (text), as a function of block type and trial type. Error bars and parenthetical figures represent 1 standard error of the mean.

Figure 2 shows overall completion times and error rates as a function of Block Type and Condition. Consistent with TMT studies, the completion time data show that having to interleave targets from both sequences is more demanding, giving rise to overall slower responses in Mixed blocks (*M* = 10.3 seconds, standard error [*SE*] = 0.5) than Sequential blocks (*M* = 7.2 seconds, *SE* = 0.34). Consistent with previous MILO studies, the Sequential block has virtually identical completion times for the Vanish (*M* = 7.2 seconds, *SE* = 0.34) and Remain (*M* = 7.1 seconds, *SE* = 0.34) blocks. The novel finding concerns the pattern of completion times for the Mixed block. Here, there is a clear additional cost in completing the trials when targets Remain (*M* = 11.0 seconds, *SE* = 0.59) compared with when they Vanish (*M* = 9.6 seconds, *SE* = 0.45). The main effects of both Block Type, *F*(1, 11) = 129.4, MSE = 0.92, *p* < .001, $\eta_{p}^{2}$ = 0.92, and Condition, *F*(1, 11) = 26.6, MSE = 0.21, *p* < .001, $\eta_{p}^{2}$ = 0.71, were qualified by a clear Block × Condition interaction, *F*(1, 11) = 32.7, MSE = 0.21, *p* < .001, $\eta_{p}^{2}$ = 0.75.

**Figure 3.**
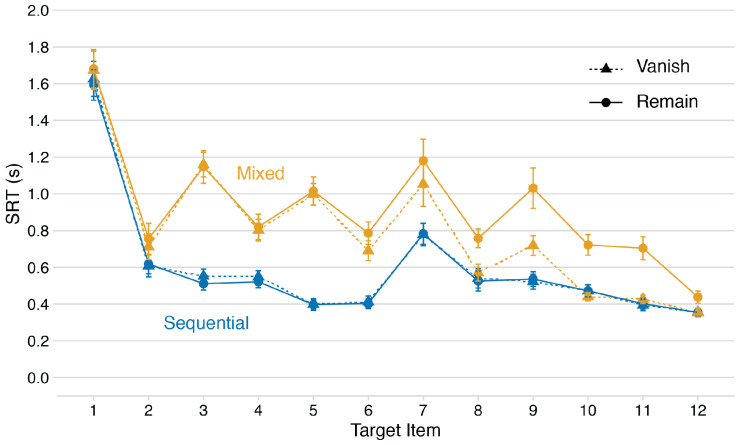
Median SRT patterns for both vanish and remain conditions as a function of block type. Error bars represent 1 standard error of the mean. SRT = serial reaction time.

The blue/darker lines in Figure 3 show SRTs as a function of Condition and Target Item for the Sequential block of trials. This pattern very closely resembles those we have observed in previous MILO studies. Specifically, there is the expected elevation of the initial response, followed by a linearly decreasing phase for subsequent items in the sequence. This is interrupted by a slower response when the target type switches, then the linear trend returns. Aside from this category switch effect, the most compelling finding from these data is the replication of the complete overlap between Vanish and Remain trials. The only significant effect was the main effect of Target Item, *F*(2.5, 27.7) = 90.9, MSE = 0.13, *p* < .001, $\eta_{p}^{2}$ = 0.89. See Supplementary Materials for more details.

The orange/lighter lines in Figure 3 show SRTs as a function of Condition and Target Item for the Mixed block of trials. It is immediately obvious that this pattern is very different from the Sequential block. Specifically, search now appears to proceed in pairs of slow-then-fast responses, giving rise to a distinctive saw-tooth pattern. The effect is amplified for the first response but is clearly visible at all other stages, except the very last two items. From the perspective of MILO, the other very interesting finding here is that SRT patterns for Vanish and Remain no longer overlap, suggesting that the additional cognitive load associated with repeated switching interferes with the ability to ignore past locations. There were main effects of both Condition, *F*(1, 11) = 33.0, MSE = 0.03, *p* < .001, $\eta_{p}^{2}$ = 0.75 and Target Item, *F*(2.7, 29.8) = 40.1, MSE = 0.29, *p* < .001, $\eta_{p}^{2}$ = 0.79, qualified by the significant Condition × Target Item interaction, *F*(5.3, 58.2) = 4.4, MSE = 0.04, *p* < .01, $\eta_{p}^{2}$ = 0.29.

In the analysis directly comparing SRT patterns in the two block types, all main effects and interactions were significant (see Supplementary Materials). Of particular note was the significant three-way Block Type × Condition × Target Item interaction, reflecting the very different patterns visible in Figure 3, *F*(4.7, 51.8) = 2.7, MSE = 0.03, *p* < .05, $\eta_{p}^{2}$ = 0.20.

## Discussion

This study used a variant of the MILO task to examine patterns of search through trials containing two sequences. We replicated the standard TMT finding that Mixed blocks (TMT-B) took consistently longer to complete, as well as the main findings of previous MILO studies—elevated first responses and overlapping Vanish and Remain curves during Sequential (TMT-A) blocks. There were also two novel findings that may help explain how increased cognitive load affects search behaviour when sequences are interleaved. We discuss these MILO findings next, before considering their implications for other tasks, such as TMT.

### Novel MILO Findings

The first novel finding is the distinctive saw-tooth pattern of within-trial SRTs during Mixed blocks (Figure 3). This pattern suggests that rather than fully alternating between the two sequences with each response (i.e., activating the full letter sequence, then the full digit sequence), participants search for successive pairs of targets (i.e., A-1, B-2, etc.), progressing in chunks of two items through both sequences in parallel. Having to repeatedly update the current search template(s) clearly has implications in terms of WM load, implications that we return to shortly.

Our suggestion is that this saw-tooth function, with slow responses followed by fast responses, provides further evidence that participants plan ahead during multiple-item search (Horowitz & Thornton, 2008; Kosovicheva et al., 2020; Thornton & Horowitz, 2004, 2020). While searching for the first member of the pair, the location of the second item is either explicitly or implicitly coded, leading to more rapid second response. This phenomenon may also be related to parallel programming of action sequences, which is known to occur for both reaching movements (Adam et al., 2000; Lavrysen et al., 2002; Vindras & Viviani, 2005) and saccades (McPeek et al., 2000; McSorley et al., 2019; Walker & McSorley, 2006).

We should note that in a previous study (Thornton & Horowitz, 2020), we did find slightly slower responses to letter sequences than digit sequences. This raises the possibility that, despite counterbalancing, the saw-tooth pattern is driven by category effects. However, this does not appear to be the case. Both in the current data set and two subsequent independent samples, we have found clear evidence of saw-tooth responding regardless of category order.

The second novel finding is that Vanish and Remain SRT patterns diverge during the more demanding Mixed blocks. Our standard finding, replicated in the Sequential blocks, is that the two SRT functions closely overlap, having an identical, accelerating profile (Horowitz & Thornton, 2008; Thornton & Horowitz, 2004, 2020). Indeed, we have argued that performance in the Remain trials provided a very compelling demonstration of how inhibitory tagging of past locations plays such an important role in everyday search and foraging behaviour (e.g., Klein, 1988; Klein & MacInnes, 1999; Wang & Klein, 2010).

The current Mixed block findings clearly indicate that increasing inherent task demands has consequences for retrospective aspects of search. The slowing of Remain responses relative to Vanish responses during Mixed blocks suggests that participants are no longer able to effectively ignore past locations and are thus not discounting those locations when searching for future targets.

Previous studies of inhibition of return have implicated a role for WM in maintaining the tagging of past locations, although such effects appear to be highly sensitive to the nature and timing of the memory tasks involved (Castel et al., 2003; Vivas et al., 2010; Zhang & Zhang, 2011). Such sensitivity may explain why previous attempts to use dual-task methodology to disrupt MILO tagging were unsuccessful (Luffingham, 2013; Zammit, 2017). Here, we speculate that some aspect of the need to maintain and dynamically update two WM search templates while moving through the interleaved sequences draws on the same resources needed for inhibitory tagging. Future MILO studies should help to further elucidate the nature of these shared resources.

### Implications for TMT and Beyond

Although our primary goal in this study was not to directly compare MILO and TMT performance, nor to champion MILO as a replacement clinical tool, the current findings clearly have implications for tasks such as TMT that involve searching through multiple targets (see also Cain et al., 2012; Hills et al., 2013; Kristjánsson et al., 2014; Pellicano et al., 2011; Wolfe et al., 2019). At the most general level, we hope we have demonstrated that examining within-trial patterns of reaction time can provide useful insights into performance, over and above examining overall completion time. While such a level of analysis is not possible with pen-and-paper tasks, computer or tablet versions of tests are likely to become more common (Fellows et al., 2017; Salthouse & Fristoe, 1995; Woods et al., 2015).

In hindsight, the idea suggested by the MILO saw-tooth patterns, that participants approach interleaved trials by chunking the sequence into matched pairs, appears obvious. However, we are not aware that this idea has been discussed in the TMT literature. If such a strategy is also used during the TMT-B, then we could attribute part of the TMT B-A difference to the demands of repeatedly and dynamically updating the current search template(s). Impairments in TMT-B performance might thus result from a compromised ability to produce consecutive *chunks* from the two sequences.

Also from the TMT perspective, we suggest that the Vanish/Remain manipulation could provide a simple way to factor out participant deficits that may be specifically associated with inhibitory control. In all current versions of TMT—whether pen-and-paper or computer-based—old targets remain visible for the duration of the test. The need to inhibit is thus confounded with other task demands. However, Figure 2 shows that Mixed block performance is significantly worse than Sequential block performance, even without the need to use inhibition (compare the two Vanish bars across block type).

When the need to inhibit is also required, during Remain trials, an additional cost is incurred, but only during Mixed blocks. The relative difference between Vanish and Remain completion times during Mixed blocks thus provides a very clear measure of the cost of having to inhibit. Importantly, such a cost can be directly measured within the task itself, without having to rely on correlational designs involving additional paradigms. Here, with normal young adults, this cost appears to be around 1.4 seconds. Introducing this simple Vanish/Remain manipulation to the TMT could provide additional diagnostic power, making it possible to identify individuals who have specific deficits with inhibitory control beyond those to be expected in matched controls. This can be done without the need to compare the full SRT functions in detail, as the difference between overall completions times in Vanish versus Remain trials would suffice.

## Conclusions

How do the inherent task demands of interleaving sequences interact with retrospective and prospective context in visual search? Using the MILO task, we found that interleaved sequences result in a characteristic saw-tooth pattern of SRTs, consistent with parallel planning for pairs of upcoming targets. Furthermore, retrospective inhibitory tagging appears to be disrupted. These findings may be relevant for research using the TMT task that inspired this experiment. Patients who experience greater difficulty in completing the TMT-B (relative to TMT-A) may have deficits in chunking future targets, inhibitory tagging, or both. Adding MILO-type manipulations may help differentiate these possibilities.

## Supplemental Material

Supplemental material for this article is available online at: http://journals.sagepub.com/doi/suppl/10.1177/2041669520958018 and https://osf.io/ugw9n/.

## References

[bibr1-2041669520958018] Adam J. J. Nieuwenstein J. H. Huys R. Paas F. G. W. C. Kingma H. Willems P. Werry M. (2000). Control of rapid aimed hand movements: The one-target advantage. Journal of Experimental Psychology: Human Perception and Performance, 26(1), 295–312. 10.1037/0096-1523.26.1.295 10696619

[bibr2-2041669520958018] Arbuthnott K. Frank J. (2000). Trail Making Test, part B as a measure of executive control: Validation using a set-switching paradigm. Journal of Clinical and Experimental Neuropsychology, 22(4), 518–528. 10.1076/1380-3395(200008)22:4;1-0;FT518 10923061

[bibr3-2041669520958018] Basoudan N. Torrens-Burton A. Jenkins A. Thornton I. M. Hanley C. Tree J. J. Thomas S. Tales A. (2019). Sequential information processing: The “elevated first response effect” can contribute to exaggerated intra-individual variability in older adults. The Yale Journal of Biology and Medicine, 92(1), 13–20. 30923469 PMC6430171

[bibr4-2041669520958018] Bowie C. R. Harvey P. D. (2006). Administration and interpretation of the Trail Making Test. Nature Protocols, 1(5), 2277–2281. 10.1038/nprot.2006.390 17406468

[bibr5-2041669520958018] Cain M. S. Vul E. Clark K. Mitroff S. R. (2012). A Bayesian optimal foraging model of human visual search. Psychological Science, 23(9), 1047–1054. 10.1177/0956797612440460 22868494

[bibr6-2041669520958018] Castel A. D. Pratt J. Craik F. I. M. (2003). The role of spatial working memory in inhibition of return: Evidence from divided attention tasks. Perception & Psychophysics, 65(6), 970–981. 10.3758/BF03194827 14528903

[bibr7-2041669520958018] Fellows R. P. Dahmen J. Cook D. Schmitter-Edgecombe M. (2017). Multicomponent analysis of a digital Trail Making Test. The Clinical Neuropsychologist, 31(1), 154–167. 10.1080/13854046.2016.1238510 27690752 PMC5286906

[bibr8-2041669520958018] Hills T. T. Kalff C. Wiener J. M. (2013). Adaptive Lévy processes and area-restricted search in human foraging. PLoS One, 8(4), e60488. 23577118 10.1371/journal.pone.0060488 PMC3618454

[bibr9-2041669520958018] Horowitz T. S. Thornton I. M. (2008). Objects or locations in vision for action? Evidence from the MILO task. Visual Cognition, 16(4), 486–513. 10.1080/13506280601087356 19730706 PMC2736545

[bibr10-2041669520958018] Klein R. M. (1988). Inhibitory tagging system facilitates visual search. Nature, 334(6181), 430–431. 10.1038/334430a0 3405288

[bibr11-2041669520958018] Klein R. M. MacInnes W. J. (1999). Inhibition of return is a foraging facilitator in visual search. Psychological Science, 10(4), 346–352. 10.1111/1467-9280.00166

[bibr12-2041669520958018] Klusman L. E. Cripe L. I. Dodrill C. B. (1989). Analysis of errors on the Trail Making Test. Perceptual and Motor Skills, 68(3_suppl), 1199–1204. 10.2466/pms.1989.68.3c.1199 2762086

[bibr13-2041669520958018] Kopp B. Rösser N. Tabeling S. Stürenburg H. J. de Haan B. Karnath H.-O. Wessel K. (2015). Errors on the Trail Making Test are associated with right hemispheric frontal lobe damage in stroke patients. Behavioural Neurology, 2015, 1–10. 10.1155/2015/309235 PMC4444530 26074673

[bibr14-2041669520958018] Kortte K. B. Horner M. D. Windham W. K. (2002). The Trail Making Test, part B: Cognitive flexibility or ability to maintain set? Applied Neuropsychology, 9(2), 106–109. 10.1207/S15324826AN0902_5 12214820

[bibr15-2041669520958018] Kosovicheva A. Alaoui-Soce A. Wolfe J. M. (2020). Looking ahead: When do you find the next item in foraging visual search? Journal of Vision, 20(2), 3 10.1167/jov.20.2.3 PMC7343403 32040162

[bibr16-2041669520958018] Kristjánsson Á. Jóhannesson Ó. I. Thornton I. M. (2014). Common attentional constraints in visual foraging. PLoS One, 9(6), e100752 10.1371/journal.pone.0100752 24964082 PMC4071029

[bibr17-2041669520958018] Lange R. T. Iverson G. L. Zakrzewski M. J. Ethel-King P. E. Franzen M. D. (2005). Interpreting the Trail Making Test following traumatic brain injury: Comparison of traditional time scores and derived indices. Journal of Clinical and Experimental Neuropsychology, 27(7), 897–906. 10.1080/1380339049091290 16183622

[bibr18-2041669520958018] Lavrysen A. Helsen W. F. Elliott D. Adam J. J. (2002). The one-target advantage: Advanced preparation or online processing? Motor Control, 6(3), 230–245. 10.1123/mcj.6.3.230 12122218

[bibr19-2041669520958018] Luffingham R. (2013). *Retrospective MILO under dual-task conditions* [BSc thesis]. Swansea University.

[bibr20-2041669520958018] McPeek R. M. Skavenski A. A. Nakayama K. (2000). Concurrent processing of saccades in visual search. Vision Research, 40(18), 2499–2516. 10.1016/s0042-6989(00)00102-4 10915889

[bibr21-2041669520958018] McSorley E. Gilchrist I. D. McCloy R. (2019). The programming of sequences of saccades. Experimental Brain Research, 237(4), 1009–1018. 10.1007/s00221-019-05481-7 30725153 PMC6430760

[bibr22-2041669520958018] Pellicano E. Smith A. D. Cristino F. Hood B. M. Briscoe J. Gilchrist I. D. (2011). Children with autism are neither systematic nor optimal foragers. Proceedings of the National Academy of Sciences, 108(1), 421–426. 10.1073/pnas.1014076108 PMC3017188 21173235

[bibr23-2041669520958018] Rabin L. A. Burton L. A. Barr W. B. (2007). Utilization rates of ecologically oriented instruments among clinical neuropsychologists. The Clinical Neuropsychologist, 21(5), 727–743. 10.1080/13854040600888776 17676540

[bibr24-2041669520958018] Reitan R. M. (1958). Validity of the trail making test as an indicator of organic brain damage. Perceptual and Motor Skills, 8(3), 271–276. 10.2466/pms.1958.8.3.271

[bibr25-2041669520958018] Salthouse T. A. (2011). What cognitive abilities are involved in trail-making performance? Intelligence, 39(4), 222–232. 10.1016/j.intell.2011.03.001 21789028 PMC3141679

[bibr26-2041669520958018] Salthouse T. A. Fristoe N. M. (1995). Process analysis of adult age effects on a computer-administered Trail Making Test. Neuropsychology, 9(4), 518–528. 10.1037/0894-4105.9.4.518

[bibr27-2041669520958018] Sánchez-Cubillo I. Periáñez J. A. Adrover-Roig D. Rodríguez-Sánchez J. M. Ríos-Lago M. Tirapu J. Barceló F. (2009). Construct validity of the Trail Making Test: Role of task-switching, working memory, inhibition/interference control, and visuomotor abilities. Journal of the International Neuropsychological Society, 15(3), 438–450. 10.1017/S1355617709090626 19402930

[bibr28-2041669520958018] Thornton I. M. Horowitz T. S. (2004). The multi-item localization (MILO) task: Measuring the spatiotemporal context of vision for action. Perception & Psychophysics, 66(1), 38–50. 10.3758/BF03194859 15095938

[bibr29-2041669520958018] Thornton I. M. Horowitz T. S. (2020). MILO mobile: An iPad app to measure search performance in multi-target sequences. i-Perception, 11(3), 1–17. 10.1177/2041669520932587 PMC7307404 32612800

[bibr30-2041669520958018] Vindras P. Viviani P. (2005). Planning short pointing sequences. Experimental Brain Research, 160(2), 141–153. 10.1007/s00221-004-1995-x 15258715

[bibr31-2041669520958018] Vivas A. B. Liaromati I. Masoura E. Chatzikallia K. (2010). Re-examining the contribution of visuospatial working memory to inhibition of return. Psychological Research, 74(6), 524–531. 10.1007/s00426-010-0274-7 20091411

[bibr32-2041669520958018] Walker R. McSorley E. (2006). The parallel programming of voluntary and reflexive saccades. Vision Research, 46(13), 2082–2093. 10.1016/j.visres.2005.12.009 16473385

[bibr33-2041669520958018] Wang Z. Klein R. M. (2010). Searching for inhibition of return in visual search: A review. Vision Research, 50(2), 220–228. 10.1016/j.visres.2009.11.013 19932128

[bibr34-2041669520958018] Wolfe J. M. Cain M. S. Aizenman A. M. (2019). Guidance and selection history in hybrid foraging visual search. Attention, Perception, & Psychophysics, 81, 637–653. 10.3758/s13414-018-01649-5 PMC6408307 30603990

[bibr35-2041669520958018] Woods D. L. Wyma J. M. Herron T. J. Yund E. W. (2015). The effects of aging, malingering, and traumatic brain injury on computerized Trail-Making Test performance. PLoS One, 10(6), e0124345 10.1371/journal.pone.0124345 26060999 PMC4465490

[bibr36-2041669520958018] Zammit T. (2017). *Location memory in visual search: The multi-item localization (MILO) task: A tool for investigation* [Master’s thesis]. University of Malta.

[bibr37-2041669520958018] Zhang Y. Zhang M. (2011). Spatial working memory load impairs manual but not saccadic inhibition of return. Vision Research, 51(1), 147–153. 10.1016/j.visres.2010.10.022 20974166

